# Respectful and disrespectful care in the Czech Republic: an online survey

**DOI:** 10.1186/s12978-018-0648-7

**Published:** 2018-12-04

**Authors:** Cecily Begley, Natalie Sedlicka, Deirdre Daly

**Affiliations:** 1 School of Nursing and Midwifery, Trinity College Dublin, 24 D’Olier Street, Dublin, DO2 T283 Ireland; 2 Institute of Health and Care Sciences, Sahlgrenska Academy, University of Gothenburg, Gothenburg, Sweden; 3 Association for Birth Houses & Centers (APODAC), Masarykovo nábřeží 234/26, 11000 Prague 1, Czech Republic

**Keywords:** Consent, Intervention, Maternity care, Labour, Obstetric violence, Respectful care, Disrespectful care, Abuse

## Abstract

**Background:**

Respectful maternity care includes treating women with dignity, consulting them about preferences, gaining consent for treatment, respecting their wishes, and giving care based on evidence, not routines. In the absence of any documented evidence, this study aimed to ascertain maternity care-givers’ perceptions of respectful care provided for childbearing women in Czech Republic.

**Methods:**

Following ethical approval, an online quantitative survey with qualitative comments was completed by 52 respondents recruited from workshops on promoting normal birth, followed by snowball sampling. The majority were midwives (50%) or doulas (46%) working in one of 51 hospitals, or with homebirths. Chi-square analysis was used for comparisons.

**Results:**

Non-evidenced-based interventions, described as ‘always’ or ‘frequently’ used in hospitals, included application of electronic fetal monitoring in normal labour (*n* = 40, 91%), shaving the perineum (*n* = 10, 29%), and closed-glottal pushing (*n* = 32, 94%). Positions stated as most often used for spontaneous vaginal births were semi-recumbent (*n* = 31, 65%) or lying flat (*n* = 15, 31%) in hospital, and upright at home (*n* = 27, 100%). Average episiotomy and induction of labour rates were estimated at 40 and 26%, respectively, higher than accepted norms.

Eighteen respondents (46%) said reasons for performing vaginal examinations were not explained to women in hospitals, and 21 (51%) said consent was ‘never’ sought. At home, 25 (89%) said reasons were explained, and permission ‘always’ sought (*n* = 22, 81%). Thirteen (32%) said hospital clinicians explained why artificial rupture of membranes was necessary, but only ten (25%) said they ‘always’ sought permission. The majority said that hospital clinicians ‘never’/‘almost never’ explained reasons for performing an episiotomy (13 = 34%), gained permission (*n* = 20, 54%) or gave local anaesthetic (*n* = 19, 51%). Contrastingly, 17 (100%) said midwives at home explained the reasons for episiotomy and asked permission. When clinicians disagreed with women’s decisions, 13 (35%) respondents said women might be told to ‘*face the consequences’*, six (16%) stated that the ‘*psychological pressure*’ experienced caused women to ‘*give up and give their permission’,* and four (11%) said the intervention would be performed ‘*against her will*.’

**Conclusions:**

Findings reveal considerable levels of disrespectful, non-evidenced-based, non-consensual and abusive practices that may leave women with life-long trauma.

## Plain English summary

An online survey which aimed to find out levels of respectful maternity care in the Czech Republic was completed by 52 respondents, mostly midwives and doulas, working in one or more of 51 hospitals or with homebirths. Practices not supported by research and described by between a third and a half of respondents as ‘always’ or ‘frequently’ used included: attaching an electronic monitor to women’s abdomen (tummy) to monitor baby’s heart in labour, shaving the area around the vagina (perineum), giving women enemas in early labour to empty the bowels and telling women to hold their breath to push when birthing. Women birthing in hospital were described as having to lie almost flat or flat in bed, but at home, all said women would be upright. Respondents said many procedures, such as vaginal examinations, breaking the waters around the baby and cutting the area around the vagina during birth were not explained to women, and over half said they were not asked for consent. When women refused treatment, staff reactions were described as threatening and, sometimes, the pressure women then experienced from staff caused them to give in and accept the treatment. One in ten respondents described treatments being done against women’s wishes.

This study showed that many women experienced care that was not supported by research, that procedures were not explained to women and that some treatments were given without consent. Many practices were not only disrespectful, but also harmful and abusive and may leave women with life-long suffering.

## Background

Respectful maternity care encompasses physical and psychological care, communication and interactions, is influenced by structural, organisational and cultural systems, and financial issues [1, 2] and implies ‘doing no harm’ [3]. The terms used to describe respectful care include both positive descriptions, such as ‘respectful’ [4, 5] and ‘humanised’ [6], and negative descriptions, such as ‘disrespectful’ [7–11], ‘obstetric violence’ [2, 12], ‘mistreatment’ [13–15] and ‘abuse’ [7, 16, 17]. Bohren et al. [15] favoured the term ‘mistreatment’, while Bowser and Hill [1] conceptualised disrespectful or abusive care in childbirth facilities (DACF) as comprising seven overlapping categories: physical abuse; non-consented care; non-confidential care; non-dignified care; discrimination; abandonment; and detention in facilities. While data on the prevalence of disrespectful treatment of childbearing women are limited [1], findings from systematic reviews leave no doubt about its existence [15, 16, 18] and levels of the inappropriate use of interventions in middle-income countries have been described as ‘worrying’ [3] (p2185).

In 2007, Venezuela became the first country formally to define and include the term ‘obstetric violence’ in legislation [12]. Respectful care in childbirth is also regarded as a human right [19] and, in 2014, the World Health Organization (WHO) acknowledged that disrespect and abuse not only violate women’s rights, but also deter women from seeking and using maternal health care services, and can have implications for their health and well-being [20]. In 2016, the second of the Lancet’s six papers on maternal health linked respectful care with evidence-based care [3], and described evidence-based maternal care as care that is ‘humane and dignified, and delivered with respect for women’s fundamental rights’ (p2178). Disrespectful intrapartum interventions, and/or interventions shown to be harmful when overused, include advising interventions to women whose labour is progressing normally and the baby is well, routine admission cardiotocography (CTG), continuous electronic fetal monitoring (EFM), decision-making in labour based on CTG findings alone, amniotomy, not encouraging women to birth in positions other than lying supine/semi-supine, episiotomies, giving enemas in labour and performing fundal pressure (Kristeller manoeuvre). Thus, evidence-based care is care based on best available evidence, and not on routines and interventions that are not clinically justified and can be harmful when used too soon or too often. Also in 2014, the International Federation of Gynaecologists and Obstetricians (FIGO) issued a working paper on Mother and Baby Friendly Birthing Facilities [21] and, with the International Confederation of Midwives (ICM), WHO and several other organisations, signed the White Ribbon Alliance’s (WRA’s) Charter on the Universal Rights of Childbearing Women [19] outlining the 10 criteria describing their essential attributes.

This paper presents findings from a survey of views of maternity care-givers (mainly midwives and doulas), on the level of respectful or non-respectful care given to women birth in the Czech Republic (CR). The study was designed after a documentary was shown on television in the CR in 2015 that appeared to show some aspects of poor practice in maternity care (https://www.youtube.com/watch?v=f-CZgwTC5bk). In some instances this was at the level of providing care that was not evidence-based, but one scenario showed clinicians applying severe fundal pressure to expedite a birth, a practice that is not based on evidence and not recommended [22]. There is little published information on rates of interventions in the Czech Republic but the  2010 EuroPeristat figures give above average episiotomy rates of 51.2%, comparatively low rates of induction of labour (10%) and pre-labour caesarean section (CS) (12.7%) and a total CS rate of 23.1% [23]. In other European countries, rates of episiotomy were 75% in Cyprus (in 2007), 68.2% in Romania, 67.5% in Poland, 36.1% in Slovenia, 16.0% in Estonia, 6.6% in Sweden and 4.9% in Denmark; rates of induced labour ranged from 33.0% in Wallonia to 8.3% in Latvia and 6.8% in Lithuania; rates of pre-labour CS ranged from 38.5% in Cyprus to 6.9% in Iceland and 6.5% in Finland, and total CS rates ranged from 52.2% in Cyprus (in 2007) to 38.0% in Italy, 32.3% in Hungary to 17.1% in both Norway and Sweden and 16.8% in Finland [23].

Attendees (*n* = 46) at recent workshops for maternity care workers in Prague spoke of instances of fundal pressure being used, and gave other examples of non-evidenced based and non-respectful care, which they believed were frequently used. This study was conducted in order to obtain maternity care-givers’ views on levels of respectful care, and their perception of the prevalence of potentially disrespectful interventions and care in the Czech Republic, in an attempt to supplement the sparse existing data.

### Maternity care in the Czech Republic

Forty years of socialist and communist regime in Czechoslovakia ended in 1989, with the Czech Republic emerging as a separate state in 1993. The repression of rights and restrictions of freedom of the whole population have, since then, been reduced and women are now becoming more autonomous and independent within what had been a very patriarchal country. However, in the maternity care environment, this progress is delayed and a hierarchical and paternalistic model of care still exists. The ideology of such a system is demonstrated in a lack of concern towards women’s needs, and autonomy [24], a lack of recognition of midwives as professionals and suppression of their expertise, including a lack of respect of autonomy. The lack of concern towards women’s needs is wide-ranging: it includes basic fundamental issues such as a lack of respect of their dignity, right to privacy during examinations and during labour to, in some instances, over-riding women’s denial of consent.

Midwifery students are taught evidence-based care, and to practise holistically, but midwifery practice within national health facilities is described as impeded by the dominance of doctors in prenatal care. Physicians also have a dominant role in antenatal care in community settings, there is no independent community midwifery care and legislation prevents midwives from providing home birth care [25].

The lack of availability of midwifery care leads to women choosing to birth alone at home, or with the support of a doula but there are no data on the number of women who choose to birth at home or birth alone. In the Czech Republic, doulas support women during pregnancy, childbirth and early motherhood (https://www.duly.cz/). During childbirth in hospital they accompany women as partners. For homebirths, if a woman is unable to find a midwife, a doula may be the only accompanying person.

## Methods

The study aimed to ascertain the views of a group of maternity care-givers, midwives and doulas, in the Czech Republic of the level of respectful or disrespectful care provided for women during pregnancy and birth. A quantitative and qualitative descriptive online survey design was used. Survey questions were based on intrapartum and postpartum evidence and recommendations from various Cochrane reviews. Ethical approval was granted by the Trinity College Dublin’s School of Nursing and Midwifery’s Research Ethics committee.

### Participants

Participants were, initially, a group of midwives and doulas working in CR who had attended at least one workshop on promoting normal birth and were willing to take part (*n* = 46). Snowball sampling was then used, which resulted in a total initial sample size of 88.

### Recruitment

A gate-keeper (PAK), a midwife who assists in running workshops to educate midwives and doulas in normal birth identified, from the database of interested participants, midwives and doulas who had shown interest in attending one of their workshops, and sent potential participants the study information packs. The information pack included an introductory letter, an information sheet and the link to the on-line survey, and was prepared in English by CB and translated into Czech language by NS and VN. Those willing to take part completed the anonymous survey forms online and had 14 days to respond, after which a reminder was resent to all potential participants by the gatekeeper. The information pack included a request to circulate the link to the survey to any other healthcare professionals that might be interested. Consent was assumed by completion and return of the online survey, and this was stated at the start of the survey.

### Data collection

The survey was anonymous, prepared in the Czech language, administered via Survey Monkey®, and included questions based on some aspects of intrapartum and postpartum evidence, and recommendations from various Cochrane reviews [22, 26–34].

Participants were asked;

(i) their profession, age range, length of time working in maternity care and their work location (hospital or home);
(ii) their hospital’s (or home birth) rates of induction and acceleration of labour, episiotomy and third/fourth perineal tears, mode of birth, frequency of neonatal birth-related injuries;
(iii) their hospital’s (or home birth) practices on offering women choice regarding induction of labour or elective caesarean section, electronic fetal monitoring, the use of local anaesthetic before suturing perineal trauma, umbilical cord clamping and cutting, skin-to-skin (SCC) contact, rooming-in and non-separation of mother and baby;
(iv) about women’s ability to move around freely during labour, positions for spontaneous vaginal birth, the practice of pushing hard on the woman’s abdomen, and/or pulling on the baby’s head, during the birth;
(v) whether or not women are informed of the reason why vaginal examination and artificial rupture of membranes are performed, if their permission is sought, and professionals’ reactions when women refuse;
(vi) if women in normal labour, with no risk factors, are permitted to drink fluids and eat a light diet in labour.

All the questions on practices were followed by free-comment text boxes.

### Data analysis

Data from the questions on the frequency with which practices occurred are presented as proportions. Statistical comparisons were made, where appropriate, using chi-square calculations [35]. Respondents’ qualitative comments are presented using direct quotations, with proportions given to illustrate the frequency of terms used.

## Findings

### Characteristics of the sample

Eighty-eight people answered the survey but 36 either entered the survey and did not answer any questions, or only completed demographic details, leaving 52 completed or partially completed surveys for analysis. Twenty-six respondents (50%) were midwives, 24 (46%) were doulas, and 2 (4%) were ‘other’ health care professionals (a psychotherapist, and a birth counsellor). The majority (*n* = 42, 81%) were aged 31 to over 40 years. Almost half of them had been working in maternity care for longer than 6 years (*n* = 24, 46%), with 11 (21%) and 16 (31%) working for 3–6 years and 1–3 years, respectively. Seven worked with home births only (14%), 6 (12%) did not wish to answer, and the remainder (*n* = 39) worked in one or more of 51 hospitals.

### Mode of birth and rates of intervention in labour

Only 11 (28%) of those who worked in hospitals said they knew the exact rates of mode of birth for their hospital, which they gave as between 20 and 32% for CS and 1.5 and 8% for instrumental birth. The remaining 41 respondents (72%) estimated rates ranging between 7 and 50% (average 26%) for CS and between 3 and 35% (average 10%) for instrumental birth.

### Rates of induction and acceleration of labour

Only 3 (6%) indicated they knew their hospital’s rates for induction of labour. The estimates made by the other respondents ranged from 15 to 50% (average 25.8%). “Overdue” was the main reason given for induction of labour (*n* = 40, 91%), defined by respondents as: *“mostly reaching [the] due date and beyond without other reason but time”*. The average timing of induction of labour was given as 41 weeks’ gestation but varied from 40^+ 0^ weeks to 41^+ 3^ weeks. One respondent stated induction of labour was performed at 42 weeks’ gestation. Estimated rates for acceleration of labour ranged from 10 to 90%, and the average rate, calculated from 20 responses, was 56%, with one respondent stating “*most – giving them oxytocin without asking – because they have applied preventively a cannula*”.

Twenty-three respondents (62%) said that women were given a choice as to whether or not they had induction of labour, and 17 (33%) respondents offered explanatory comments such as: “[women]…*have choice* [but are] *put are under big pressure so they give up and let them be induced…”*, “*…women have* [an] *option officially but… …in reality, they are often threatened that if they don’t accept induction, they will put the baby’s health in danger…”* and *“…in theory they* [women] *might choose but* [only after] *manipulative threatening negotiation”.* Five (15%) said that women in their hospital had a choice as to whether or not they had an elective CS, and six others commented that it could happen unofficially, one of whom said “*officially maybe not, but there is always [a] way to do it*.”

### Rates of episiotomy and third/fourth degree perineal tears

Similarly, only 2 (6%) knew their hospital’s rates for episiotomy and third/fourth degree tears. Estimated episiotomy rates varied mostly from 20 to 90% (with 2 responses of 2%), giving an average of 40%. Respondents caring for women in home settings stated they did not perform episiotomies.

### Intrapartum interventions always or frequently used

In hospital, the following interventions were described as being used ‘always or ‘frequently’: directed, closed-glottal pushing (*n* = 32, 94%); pushing back the cervix when it is nearly, but not fully, dilated (*n* = 11, 33%); offering drugs to relieve pain (*n* = 22, 67%); tying the woman’s legs up in stirrups (*n* = 2, 6%); shaving the perineum (*n* = 10, 29%) giving an enema in early labour (*n* = 20, 59%); asking questions and taking down details while the woman is having a contraction (*n* = 17, 52%); routine cannulation to give intravenous fluids (with no reason) (*n* = 16, 49%). At home, none of the above interventions were ‘always’ used and only one (asking questions and taking down details while the woman is having a contraction) was said by one respondent to be used frequently.

Pulling hard on the baby’s head to get him/her out, without waiting for the next contraction and for rotation of the head to occur, was said to occur ‘frequently’ (*n* = 16, 47%) or ‘sometimes’ (*n* = 12, 35%) in hospital births. In contrast, in relation to home births, only one midwife (5%) said that such efforts would be made ‘sometimes’.

### Care in normal labour

Forty respondents (91%) said that electronic fetal monitoring (EFM) was applied frequently in normal labour without a clear indication. A large majority (*n* = 27, 64%) said that when EFM was applied, women were informed of the reason why it was needed. However, 18 respondents (45%) commented that EFM was regarded as part of basic care, and only 13 (31%) said that the women were given a choice as to whether or not they accepted it. Eighteen respondents made comments such as “*CTG is taken as basis for care of all women*,” “*no asking, no option, no discussion*,” “*you have to have this*”. If women refused, “*manipulative treatment*” was instituted and if refusal continued a “negative reverse” form had to be signed, indicating that they had refused recommended treatment.

Once electronic monitoring was applied, 29 respondents (71%) said that women took up various positions in bed (lying flat, lying on their side, sitting upright), while 4 (10%) said they had to lie flat, and 8 (20%) said they could mobilise freely. Thirty-five respondents (88%) said that all women in hospital could move around freely in labour as did all 34 who had knowledge of home births.

### Performing vaginal examinations intrapartum

When asked whether or not the midwife or doctor in hospital would explain to women why they thought it was necessary to carry out a vaginal examination, 18 (46%) said ‘no’ and 8 (21%) said ‘yes, always’. In addition, 21 (51%) said clinicians in hospital would never ask the woman’s permission to do the examination, and six (15%) stated that they would inform the woman of what they were going to do. Of those who cared for women at home, 25 (89%) said that they always explained to women the necessity for carrying out a vaginal examination, and 22 (81%) ‘always’ and 5 (19%) ‘sometimes’ asked her permission. These described differences between hospital and home care are statistically significant (chi-sq = 32.06, d.f. = 2, *p* < 0.0001 (explaining) and chi-sq = 32.9, d.f. = 2, *p* < 0.0001 (asking permission).

### Performing artificial rupture of membranes (amniotomy)

Thirteen respondents (32%) stated that the midwife or doctor in hospital would explain to women why they thought it was necessary to carry out artificial rupture of membranes (ARM) and a further 24 (59%) would sometimes explain. Twelve respondents (30%) also commented that women were told *“…it’s dangerous to leave it* [amniotic membranes] *intact…”* or that *“it’s* [ARM] *necessary”* and eight respondents (20%) stated that women were told that ARM was *“…necessary to speed up the labour”.* However, only 10 (25%) said that clinicians would ‘always,’ and 19 (48%) would ‘sometimes,’ ask the woman’s permission. At home, 15 midwives (88%) would ‘always’ and 1 (6%) would ‘sometimes’ explain why it was necessary to carry out ARM, and 14 (82%) and 2 (12%) would ‘always’ and ‘sometimes’ ask permission. These statements comparing differences between hospital and home care were also statistically significant (chi-sq = 15.89, d.f. = 2, *p* < 0.001 (explaining) and chi-sq = 16.1, d.f. = 2, p < 0.001 (asking permission)).

### Eating and drinking in labour

All women labouring at home, with no high-risk factors, were permitted to drink fluids in labour, and eat a light diet such as yoghurt, soup, bread, biscuits or fruit. Fewer respondents said that similar women in hospital were allowed to drink fluids (*n* = 39, 95%), a non-significant difference, and eat light diet (*n* = 31, 76%), a statistically significant difference (Yates’ chi-sq = 6.86, d.f. = 1, *p* < 0.01).

### Clinicians’ reactions to women’s refusal of treatment or intervention

Respondents were asked: “When women refuse any treatment or intervention offered in hospital, what is the midwife’s, doctor’s or doula’s reaction?” Fourteen (37%) of the 38 respondents described the midwives’, doctors’ or doulas’ reactions as *“blackmailing”* or “*threatening*”, that they *“frighten*[ed]”, *“reject*[ed]”, *“accused*” or *“*[put] *pressure*” on women, became *“aggressive*” or *“reacted emotively*”. Respondents stated that women were *“threatened, undergo negative or unpleasant reactions and are … manipulated”,* that they were told that *“their baby is being endangered”* or that their *“decision is putting your baby at risk of death*”. Eleven (29%) described clinicians as being “*argumentative*” and that they “*attempted to convince*” women to accept the intervention. Clinicians were described as “*upsetting* [women]”, “[displaying] *arrogance*”, “[becoming] *distant*”, “[being] *pushy*,” “*forcing* [women],” with some “*calling the head obstetrician*”.

Ten respondents (26%) said clinicians would “*explain the benefits*” “[of the intervention or treatment]” and while they were “*sometimes not happy*” with the woman’s decision, they were “*accepting”* and “*respected her decision*”. Three respondents (8%) stated that clinicians’ reactions depended on the situation, and any of the aforementioned reactions might occur.

### Outcomes for women when clinicians disagreed with women’s refusal of treatment or intervention

Thirteen (35%) of the 37 respondents stated that if “*the woman defends and holds* [on to] *her position* [and] *her decision*”, she might be asked to sign a “negative reverse” (waiver of responsibility for the hospital) and be told to “*face its consequences*”. In addition, women “*might experience an unpleasant atmosphere or* [be made feel] *fearful*”, that clinicians may “*put emphasis on harming* [the] *baby*” and that their attitude towards women might change, and they may display intolerance.

Six respondents (16%) stated that the “*psychological pressure, fear and coercion*” women experienced caused them to “…*resign, give up and give their permission to perform procedure*”. A further four (11%) stated that “*pressure is strong and manipulative, women do not get choice and* [the] *intervention is performed without discussion or asking* [her], *or against her will*”. Twelve respondents (33%) stated that, depending on the specific situation, any one of the above outcomes might arise. Just two (5%) stated that the will and wish of women was respected.

### Positions for labour and birth

The position most used in hospital for women having a spontaneous vaginal birth was semi-recumbent (on a bed or couch propped up with a back-rest or pillows) (*n* = 31, 65%) or lying flat with one or two pillows under her head (*n* = 15, 31%). This contrasted with the positions used most often at home, which were upright (standing, squatting, kneeling, on all fours, on a birthing stool) (*n* = 27, 100%). These statements comparing positions used in hospital and at home showed a statistically significant difference (chi-sq = 63.79, d.f. = 1, < 0.001).

### Pushing on the woman’s abdomen or pulling the baby’s head during vaginal births

Respondents were asked how often, in their view, midwives or doctors pressed hard on a woman’s abdomen to push the baby out, during spontaneous or instrumental birth. In relation to hospital births, 13 (45%) said ‘in about a quarter of all births’ and 9 (31%) said ‘in about half of all births’. The answer in relation to home births, from 23 respondents (96%), was ‘never’. Twenty-two respondents (63%) said that the clinicians in hospital would explain to the woman why they thought this was necessary and 6 (18%) said that they would ask the woman’s permission.

### Performing episiotomy and using local anaesthetic prior to suturing perineal trauma

Respondents were asked if, when the midwife or doctor in hospital was about to perform an episiotomy, they did explain the reason to the woman, ask her permission, and give a local anaesthetic beforehand. Thirteen (34%) said the clinicians ‘never’ or ‘almost never’ explained the reason, 20 (54%) said they ‘never’ or ‘almost never’ asked permission and 19 (51%) ‘never’ or ‘almost never’ gave a local anaesthetic. Thirty-two (84%) said that the clinician would always give a local anaesthetic before suturing, if one had not been given before. In contrast, when asked about home births (where episiotomy was very seldom performed), 17 (100%) said that the midwife would explain the reason for the episiotomy and ask permission, but 8 (53%) said that local anaesthetic would ‘never’ or ‘almost never’ be given, similar to the results in hospital births. However, 15 (100%) said that a local anaesthetic would then always be given before suturing the perineum.

### Newborn outcomes and care

Respondents were asked how often they would see a baby with any diagnosed injuries from birth in hospital (e.g., bruises, fractured bones, nerve damage, paralysis). Nine (27%) gave figures of between one in every 10 to 30 births and 18 (55%) said ‘occasionally’. Twenty (95%) respondents said they had never seen such injuries after a home birth. In hospital, it was reported by 50% of respondents that the baby’s cord was either clamped or cut immediately (*n* = 6), or after one minute (*n* = 10). Eight respondents (25%) said that the cord was left unclamped for 2–3 min, and a further 8 (25%) said it was left until pulsation ceased. For births at home, 100% (*n* = 23) said the cord was left until it stopped pulsating.

### Skin-to-skin care

After hospital births, it was reported that babies were frequently placed ‘skin-to-skin’ with their mothers immediately after birth (*n* = 17, 50%) or after the baby was weighed and measured (*n* = 15, 47%). For babies who were given to their mothers immediately, however, 22 respondents (67%) said they were only left for up to 5–20 min, with 5 (15%) saying they were left over an hour. For those babies who were weighed first, ten respondents (35%) said they were only left for up to 5–20 min with their mothers, and 9 (31%) said they were then left over an hour. After home births, all respondents (*n* = 24) said babies were frequently placed ‘skin-to-skin’ immediately after birth, for more than an hour.

### Mother-baby separation postpartum

Babies were said to stay ‘frequently’ beside their mothers at all times while they were in hospital, by 13 respondents (39%), or ‘sometimes’ by 16 (49%). Four respondents (12%) said this ‘almost never’ occurred.

### Support for home births

Respondents were asked if, in their view, women had easy access to professional support for home births. Two (6%) believed they did, but the majority (*n* = 33, 94%) said they did not.

## Discussion

Miller et al. (2016) [3] describe the existence of two extremes on the continuum of maternal health care; too little, too late (TLTL) and too much, too soon (TMTS), the latter describing non-evidence-based interventions and the routine over-medicalisation of normal pregnancy and birth, which constitutes disrespectful maternity care [1]. Some of the practices reported by these respondents could be listed in more than one of Bohren et al’s typology themes [15]. Respondents’ reports of care and interventions used ‘always’ or ‘frequently’ are examined here alongside current evidence, to determine whether or not they are evidence-based, consensual or non-consensual, fall below professional standards, likely to cause pain, distress or harm and physical abuse. Very few respondents knew their hospital’s rates of mode of birth, induction and acceleration of labour, or episiotomy/third or fourth degree perineal trauma, as their hospital did not make public such data. This means that local hospital rates cannot be compared with, or benchmarked against, national or international rates, nor can one completely rely on published national rates.

In 2010, the Czech Republic’s induction of labour rate was reported as being under 10%, the pre-labour caesarean section rate was 12.7%, and the spontaneous onset of labour was 77.3%, instrumental birth rates were less than 2%, rates which, if accurate, compare favourably with other European countries [23]. However, in women birthing vaginally, the episiotomy rate was 51.2% and rates of third and fourth degree perineal trauma are not reported [23]. These respondents’ estimates (drawn from working in 51 hospitals) of 26% for induction of labour, 26% for CS, and 10% for instrumental birth rates are far higher than those in official statistics, perhaps indicating some inaccuracies in record-keeping.

### Evidence-based care

Several practices reported as being ‘always’ or ‘frequently’ used in hospitals were not evidence-based, and there were statistically significant differences between statements on home and hospital practices in relation to eating and drinking in labour and positions for labour and birth. Only 76% said women labouring in hospitals were permitted to eat a light diet. Restricting fluids and food in labour for women at low risk of complications is not justified [34], and may contribute to the use of intravenous fluids unnecessarily, a practice which, according to 49% of respondents occurred ‘always’ or ‘frequently’ in hospital. The vast majority said closed-glottal pushing during the second stage of labour was ‘always’ or ‘frequently’ used in hospitals, which has been shown in small studies to affect urodynamic factors negatively at 3 months postpartum [36], and to increase some adverse neonatal outcomes [37], although others have found no difference [38]. A Cochrane review has stated that while the evidence to support or refute any particular pushing style is inconclusive, in the absence of strong evidence, the woman’s preference, comfort and clinical context should guide decisions [38].

Almost one third of respondents said women’s perineums were ‘always’ or ‘frequently’ shaved in hospitals, a practice that has no clinical benefit and should never be performed [39]. Indeed, all of the clinical trials included in this review were conducted pre 2005, reflecting the belief that shaving the perineum is unnecessary and not shaving the perineum is safe has been accepted for the past 15 years [40]. Almost two-thirds of respondents said women were ‘always’ or ‘frequently’ given enemas in early labour, a practice that should be not be performed [41]. In hospitals, almost half of respondents said that EFM was ‘frequently’ used in normal labour without a clear indication. While EFM during labour is associated with a reduction in neonatal seizures rates (albeit with no long-term benefit to the babies), it is associated with an increase in caesarean sections and instrumental vaginal births, and should not be used routinely in normal labour [26]. When EFM was used, only 20% of respondents said women could mobilise freely; the majority said women adopted various positions in bed or had to lie flat, practices that restrict women’s mobility, their ability to adopt upright positions, and do not facilitate labour progress [31].

Respondents said the umbilical cord stopped pulsating before being clamped or cut in all home births. In hospital, 50% of respondents said that the baby’s cord was either clamped or cut immediately, or after 1 min. In healthy term infants, the evidence states that a more liberal approach to delaying clamping of the umbilical cord is beneficial to neonates [32].

Almost all respondents said that babies were frequently placed in skin-to-skin contact (SSC) with their mothers immediately after birth, or after being weighed and measured, in hospital births. However, two-thirds also said that babies given to their mothers immediately were only left with them for 5–20 min and, when babies were weighed first, one-third said they were only left with their mothers for 5–20 min. In contrast, all respondents said babies born at home were frequently placed in SSC immediately after birth, for more than an hour. The evidence supports using immediate or early SSC to promote breastfeeding, and concludes that early SSC should be normal practice for healthy babies [33].

### Practices which fall below professional standards of care

Almost two-fifths of respondents said babies frequently, half said they sometimes, stayed beside their mothers in hospital at all times and more than one in ten said babies never stayed beside their mothers in hospital. There is little evidence to support or refute the practice of rooming-in versus mother-infant separation, with the one review identified including only one trial with 176 participants [29], so further well-designed trials are needed. However, all women who wish their babies to stay beside them while in hospital should be facilitated.

### Consensual and non-consensual care

The WHO’s (2016) quality statement number 5.3 states that *‘All women have informed choices in the services they receive, and the reasons for interventions or outcomes are clearly explained’* [42]. In this study, 46% said the midwife or doctor in hospital would not explain why a vaginal examination was necessary, and 51% said women’s consent was never sought. At home, the majority said the reasons for performing the vaginal examination were explained, and permission was always sought. Worryingly, permission to perform the examination was not sought from all women in all settings, meaning that the care some women receive not only falls below acceptable standards, but also is unethical and tantamount to physical and sexual abuse [15]. Similarly, only one-third of respondents said that clinicians in hospitals explained why ARM was necessary, and only one-quarter said they ‘always’ sought permission.

In home births, many stated that episiotomies were never performed but all respondents said that, on the rare occasions when an episiotomy was done, the midwife would explain the reason, and ask women’s permission. In contrast, one-third said clinicians in hospitals ‘never’ or ‘almost never’ explained the reason why episiotomies were performed. More than half said clinicians ‘never’ or ‘almost never’ sought permission, despite episiotomy being a surgical incision in a sensitive area of the body.

In relation to fundal pressure to expel the baby during vaginal birth, while 63% said the reason for this was explained, only 18% said that clinicians would ask the woman’s permission. Women cannot give consent to treatments, procedures or interventions unless they are informed, and not gaining consent is disrespectful and abusive [1]. In particular, practices such as performing vaginal examinations, ARMs, episiotomies and fundal pressure without informed consent are types of physical and sexual abuse [16].

### Practices that are likely to cause pain, distress or harm

Some of the non-evidence-based practices described above were also potentially or actually harmful. In hospitals, one-third of respondents also described clinicians pushing back the cervix when it was almost, but not fully, dilated. The cervix is a highly sensitive circular muscle with an abundance of nerve fibres, and it is highly likely that such practice causes women considerable avoidable pain. Just under half of respondents said pushing on the woman’s abdomen occurred in about a quarter, and 31% said in about half, of all births in hospital. The majority said this practice never happened in home births. While there is insufficient evidence to support the use of fundal pressure by any method in the second stage of labour, none of the nine included studies reported on possible severe problems or death of the women [22]. It is certain, however, that this practice causes women pain, often severe. In addition, half of respondents said that women in hospital and at home would ‘never’ or ‘almost never’ be given local anaesthetic prior to performing an episiotomy, so this seems to be a widespread practice.

The majority of respondents said pulling hard on the baby’s head to get him/her out without waiting for the next contraction and for rotation of the head, occurred ‘frequently’ or ‘sometimes’ in hospital births, and only one midwife said that such efforts occurred sometimes in home births. There is no evidence to support this practice, a practice which interferes with the physiological mechanism of birth and can inflict serious trauma to the baby. While we could not look at associations between this practice and birth injuries, 27% of respondents said they saw babies with diagnosed birth injuries in one in every 10 to 30 hospital births, and 55% saw such injuries occasionally. The majority never saw such injuries in home births.

### Physical abuse

Two respondents said women in hospital would have their legs tied up in stirrups, a practice that is both restraining and physically abusive [1, 15] and inconsistent with the WHO’s (2016) [42] quality statement 5.2 which states ‘*No woman or newborn is subjected to mistreatment, such as physical, sexual or verbal abuse…’*. Thirty-two respondents (84%) said that the clinician in hospital would always give a local anaesthetic before suturing the perineum, if one had not been given before. In home births all respondents said that a local anaesthetic would always be given before suturing. Estimated episiotomy rates were high, despite the fact that episiotomy does not reduce perineal/vaginal trauma and is not justified routinely [30]. Performing an episiotomy without consent is an assault, and performing it without administrating pain relief, or suturing the perineum without pain relief, is not acceptable. The other practices discussed under “non-consensual care” are also instances of physical, or sexual, abuse.

### Access to professional support for home birth

The majority of respondents said women did not have easy access to professional support for home births. This means women have limited options, especially if they reject care in hospital because of previous experiences. In the absence of easy access to professional support for home birth, women may choose to birth without professional assistance because of dissatisfaction with hospital birth [43], previous traumatic experiences [44] and a desire to avoid unnecessary interventions [43–47].

### Strengths and limitations

There is a relative lack of formal research around respectful, non-abusive birth care [1] and a major strength of this study is that it presents, for the first time, 52 respondents’ descriptions of intrapartum maternity care practices in both hospital and home birth settings in the Czech Republic. The sample for this study is necessarily self-selecting, and may therefore be biased. However, while the respondents’ opinions are not necessarily representative of all practitioners, and the findings cannot be generalised, they nevertheless report on the care provided in 51 hospitals (50% of the total in CR) and reveal the widespread use of practices that are not only not evidence-based, but also cause harm and/or are physically, emotionally or sexually abusive. Much of the data is based on estimates, as hospitals in CR do not routinely publish their annual figures, but the fact that some abusive, or non-evidence-based, practices exist at all is important for noting. Further research can then be done officially in the CR, to determine the actual rate of these practices.

### Implications for practice

The findings from this study present a ‘snapshot’ of hospital and home birth practices in the Czech Republic in 2016/2017. They reveal the widespread use of many interventions which, in the absence of clinical need, represent disrespectful care, and some interventions that are not evidence-based and/or are harmful and contribute to maternal and newborn morbidity; this indicates a lack of general national standards and guidelines for providing evidence-based maternity care in the CR.

It is important to note that mistreatment or abusive conduct by health care providers is not necessarily intentional, and may coexist with other compassionate and respectful care practices, however, such practices may leave women with life-long physical and emotional trauma [48, 49]. Regardless of intent, only women’s own experiences and their feelings about mistreatment should be viewed as conclusive in terms of the trauma suffered. A companion study, inviting all childbearing women in the CR to respond, commenced in February 2018.

At a minimum, respectful maternity care requires that women are informed about clinically necessary procedures, and give consent. While prevalence of disrespect in health facilities was not estimated because of the lack of a validated measurement tool and lack of operational definition [1], a more recent study across six European countries reported prevalence of experienced abuse at 20.7% [17]. While the reasons for disrespecting or abusing women are poorly understood, the consequences are clear: disrespectful and/or abusive care deter women from seeking the help of skilled professionals [1, 45]. Further research is required into many aspects of disrespectful care, particularly in those countries not yet studied, including the extent and impact, the ways in which it deters women from seeking skilled professional care and strategies to eliminate its existence.

## Conclusion

The findings from this study reveal practices in the Czech Republic that are not only not evidence-based, but also are non-consensual and abusive to women. Such practices may leave women with life-long physical and emotional trauma. At a minimum, no procedure should be performed without women’s expressed consent and authority and, in the words of FIGO [21] ‘*Every woman and every baby should be protected from unnecessary interventions, practices and procedures that are not evidence-based, and any practices that are not respectful of their culture, bodily integrity, and dignity*’ (p1). In other words, every childbearing woman should receive evidence-based and respectful attention, every time; that is the minimum expected level of maternity care everywhere.

## Abbreviations

ARM: artificial rupture of membranes
EFM: electronic fetal monitoring
SCC: skin-to-skin care
